# Antioxidative Activity of Ferrocenes Bearing 2,6-Di-*Tert*-Butylphenol Moieties

**DOI:** 10.1155/2010/165482

**Published:** 2010-06-09

**Authors:** E. R. Milaeva, S. I. Filimonova, N. N. Meleshonkova, L. G. Dubova, E. F. Shevtsova, S. O. Bachurin, N. S. Zefirov

**Affiliations:** ^1^Organic Chemistry Department, M.V. Lomonosov Moscow State University, 119991, Moscow, Russia; ^2^Institute of Physiologically Active Compounds, Russian Academy of Sciences, 142432, Chernogolovka, Russia

## Abstract

The antioxidative activity of ferrocenes bearing either 2,6-di-*tert*-butylphenol or phenyl groups has been compared using DPPH (1,1-diphenyl-2-picrylhydrazyl) test and in the study of the in vitro impact on lipid peroxidation in rat brain homogenate and on some characteristics of rat liver mitochondria. The results of DPPH test at 20°C show that the activity depends strongly upon the presence of phenolic group but is improved by the influence of ferrocenyl fragment. The activity of N-(3,5-di-*tert*-butyl-4-hydroxyphenyl)iminomethylferrocene (**1**), for instance, was 88.4%, which was higher than the activity of a known antioxidant 2,6-di-*tert*-butyl-4-methylphenol (BHT) (48.5%), whereas the activity of N-phenyl-iminomethylferrocene **2** was almost negligible −2.9%. The data obtained demonstrate that the compounds with 2,6-di-*tert*-butylphenol moiety are significantly more active than the corresponding phenyl analogues in the in vitro study of lipid peroxidation in rat brain homogenate. Ferrocene **1** performs a promising behavior as an antioxidant and inhibits the calcium-dependent swelling of mitochondria. These results allow us to propose the potential cytoprotective (neuroprotective) effect of ditopic compounds containing antioxidant 2,6-di-*tert*-butylphenol group and redox active ferrocene fragment.

## 1. Introduction

Oxidative stress has been found to play a critical role in numerous disease conditions including neurodegeneration [1–3]. 

The antioxidative defense system in living organism regulates a disturbance in the prooxidant-antioxidant balance and protects the cell damage induced by high level of oxidative stress. Among the classes of well-known natural antioxidants-vitamins E group, ascorbic acid, glutathione, and so forth, *α*-tocopherol and its synthetic analogues, sterically hindered phenols, are of particular importance [4]. The substituted 2,6-dialkylphenols are widely used as inhibitors of free radicals formation in the oxidative destruction of natural and synthetic substrates. The mechanism of their physiological action is associated with the stable phenoxyl radicals' formation in the process of hydrogen atom abstraction by highly reactive peroxyl radicals of lipids [5]. 

The goal of this study was to optimize the effect of 2,6-di-*tert*-butylphenol and to increase the stability of the corresponding phenoxyl radicals responsible for their antioxidative activity. The approach based on modification of phenolic antioxidants *via* incorporation of ferrocenyl moiety in their molecules seems to be a promising one. Previously we have reported the synthesis, electrochemical characteristics, and ESR study of novel ferrocenes with redox active 2,6-di-*tert*-butylphenol fragments (compounds **1**,**3**) [6]. These compounds exhibit the properties of multistep redox systems, and the intramolecular electron transfer between two redox active sites of the molecule (the phenol and ferrocene groups) was observed. The high stability of phenoxyl radical species formed in the oxidation is in agreement with a certain degree of electronic delocalization over the molecule.

On the other hand, the ferrocene derivatives show a wide spectrum of physiological activity [7–9].

The incorporation of ferrocene into an anticancer drug tamoxifen, a selective estrogen receptor modulator, containing phenol was reported. The activity of these novel ferrocene derivatives (ferrocifens) was found to be associated with the proton-coupled electron transfer between ferrocenium ion and phenol group that occurs in their oxidized species [10–12].

The antioxidative activity in scavenging of superoxide radical-anion O_2_^•−^ and HO^•^ radical was observed for recently synthesized ferrocenes containing nitroxides radicals as substituents [13]. 

As it has been reported earlier, diselenides having redox-active ferrocenyl units show peroxidase-like antioxidant activity mimicking selenoenzyme glutathione peroxidase that protects the cell membranes from oxidative damage [14].

In our previous study, we have observed the modulation of the antioxidative effect of metalloporphyrins bearing 2,6-di-*tert*-butylphenol pendants by the metal nature [15].

**Figure 1:**
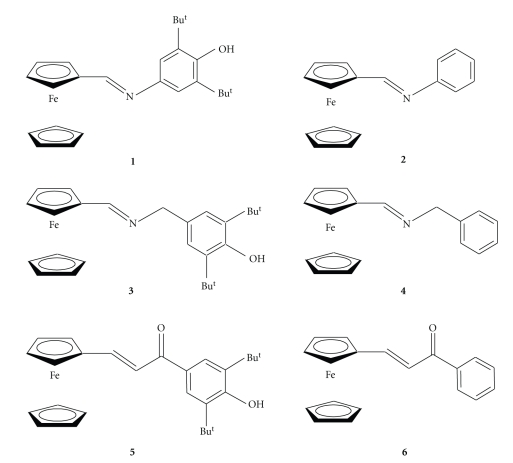
Structures of compounds **1**–**6**.

In this study we compared the antioxidative activity of **1**–**6** presenting the pairs of compounds bearing either 3,5-di-*tert*-butyl-4-hydroxyphenyl or phenyl substituents linked to the ferrocene by various spacers (Figure 1).

## 2. Materials and Methods

### 2.1. Ferrocenes

N-(3,5-di-*tert*-butyl-4-hydroxyphenyl)-iminomethylferro-cene (1), N-phenyl-iminomethylferrocene (2), N-(3,5-di-*tert*-butyl-4-hydroxybenzyl)-iminomethylferrocene (3), N-benzyliminomethylferrocene (4), (3,5-di-*tert*-butyl-4-hydroxyphenyl)-3-ferrocenylpropen-2-on (5), and phenyl-3-ferrocenylpropen-2-on (6) were synthesized as described previously [6, 16]. 

### 2.2. DPPH Radical Scavenging Activity

The free radical-scavenging activity was evaluated using the stable radical DPPH, according to the method described by Brand-Williams et al. [17] with a slight modification.

Each compound was tested for antioxidant activity against DPPH radical at a molar 1 : 1 ratio. One mL of antioxidant solution in methanol was added to 1 mL of DPPH solution in methanol so that the final DPPH and antioxidant concentration can be 0.1 mM. The samples were incubated for 30 minutes at 20°C in methanol and the decrease in the absorbance of DPPH solution was measured at 517 nm, using a Thermo Evolution 300 BB spectrophotometer. The results were expressed as scavenging activity, calculated as follows:

(1) $$\text{Scavenging}\;\text{activity},\text{\%}\;=\;\left[\frac{\left(A_{0}-A_{1}\right)}{A_{0}}\right]\;\times \;100.$$

The concentration of antioxidant needed to decrease 50% of the initial substrate concentration (EC_50_) is a parameter widely used to measure the antioxidant effect [18]. For determination of EC_50_, the values of DPPH solution absorbance which decrease after 30 minutes were used. The EC_50_ values were calculated graphically by plotting scavenging activity against compound concentration. Different sample concentrations (0.01, 0.02, 0.05, and 0.1 mM) were used in order to obtain kinetic curves and to calculate the EC_50_ values. The lower EC_50_ means the higher antioxidant activity. 

### 2.3. Rat Brain Homogenates (RBH) and Rat Liver Mitochondria (RLM) Preparation

On the day of the experiment, adult Wistar male rats fasted overnight were euthanized in a CO_2_-chamber followed by decapitation. The procedure was in compliance with the Guidelines for Animal Experiments at Institute of Physiologically Active Compounds of Russian Academy of Sciences.

The brains were rapidly removed and homogenized in 0.12 M HEPES/0.15 M NaCl, pH 7.4 buffer (HBS) (10 mg/gr wet weight) and used immediately for assay. 

Mitochondria were isolated from homogenates of livers of adult Wistar strain rats, fasted overnight, in a 5 mM HEPES buffer, pH 7.4, containing 210 mM mannitol, 70 mM sucrose, and 1 mM EDTA, by conventional differential centrifugation [19]. 

Protein concentrations in RBH and RLM were determined by the biuret assay using bovine serum albumin as a standard [20].

### 2.4. *F* *e* ^3+^-Induced Lipid Peroxidation Assay

The extent of lipid peroxidation (LP) was estimated by the levels of malondialdehyde measured using the thiobarbituric acid reactive substances (TBARS) assay. Isolated mitochondria are metabolically active and tightly coupled as shown by respiratory control ratio values, which were about 4 with glutamate-malate as substrate as measured by mitochondrial oxygen consumption at Oroboros oxygraph (Anton Paar, Austria) in a medium containing 10 mM KH_2_PO_4_ (or NaH_2_PO_4_), 60 mM KCl, 60 mM Tris, 5 mM MgCl_2_, 110 mannitol, and 0.5 mM EDTA-Na_2_, pH 7.4.

Study of compounds influence on LP of the RBH was carried out at 30°C for 40 minutes in 0.25 mL of the RBH in HBS (2 mg of protein · mL^−1^) in the presence or absence of compounds or vehicle (DMSO). LP was induced by using Fe^3+^ (0.5 mM Fe(NH_4_)(SO_4_)_2_) as an oxidizing agent [21]. Then 0.25 mL aliquots were mixed with 0.5 mL thiobarbituric acid (TBA) medium containing 250 mM HCl, 15% trichloroacetic acid, and 3 mM TBA, heated at 95°C for 15 minutes, cooling at 4°C then probes centrifuged (10 minutes at 10 000 g) and the supernatants transferred into 96-plate and absorbance was measured at 530–620 nm at the Wallac Victor 1420 Multilabel Counter (PerkinElmer Wallac). 

All the experiments were performed using four independent experiments with different brain homogenate preparations. Data are normalized to control probe with oxidant as 100% and blank probe with diluent but without oxidizing agent. Preliminary experiments were done in the absence of compounds interaction with thiobarbituric acid. The values are expressed as mean%  ± SD. The concentrations of ferrocenes giving half-maximal inhibition (IC_50_) of LP were determined by dose-effect analysis.

### 2.5. Mitochondrial Swelling Assay

Mitochondrial swelling caused by influx of solutes through open mitochondrial permeability transition (MPT) pores results in an increase in light transmission (i.e., a reduced turbidity). This turbidity change offers a convenient and frequently used assay of the MPT by measurement of absorbance in mitochondrial suspensions. The MPT induced by Ca^2+^ was monitored by absorbance changes at 540 nm in a Beckman DU 640 spectrophotometer in 1 mL of buffer A plus 0.8 *μ*M rotenone, 5 mM succinate, 1 mM KH_2_PO_4_, and 0.5 mg protein of isolated liver mitochondria at 30°C and continuous stirring [19]. Swelling rate is quantified as ΔA_540_/min/mg, calculated, in all cases, from a tangent to the steepest portion of the plot of A_540_ versus time.

### 2.6. Measurement of Mitochondrial Membrane Potential

The same experimental conditions were used for the assessment of alterations of the mitochondrial membrane potential, except that safranine was included in incubation medium at a final concentration of 10 *μ*M and succinate was added after the compound. This concentration of safranine was determined before hand as the optimal compromise between signal/baseline ratio and interference of safranine itself with swelling induced by Ca/Pi (safranine tended to enhance Ca/Pi-induced swelling at concentrations above 20 *μ*M) [19]. Changes in the status of the MPT pore are assessed spectrophotometrically at 524 versus 554 nm in a Beckman DU 640 spectrophotometer at 30°C and continuous stirring.

## 3. Results and Discussion

We have compared the antioxidative activity of **1**–**6** presenting the pairs of compounds bearing either 3,5-di-*tert*-butyl-4-hydroxyphenyl or phenyl substituents linked to the ferrocene by various spacers (Figure 1). 

**Figure 2:**
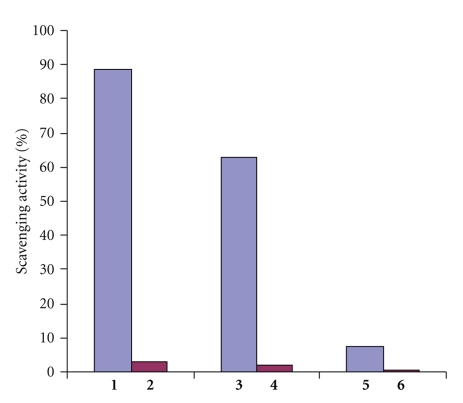
Scavenging activity for compounds **1**–**6** in DPPH test (MeOH, 20°C, 100 *μ*M).

The scavenging activity has been studied in the process of hydrogen atom transfer to the stable free radical DPPH [22]. The results of DPPH test at 20°C show that the activity depends strongly upon the presence of phenolic group in the presented pairs of compounds. The activity of **1**, for instance, was 88.4% that is higher than that of a known antioxidant 2,6-di-*tert*-butyl-4-methylphenol (BHT) (48.5%) whereas the activity of **2 **bearing phenyl substituent was almost negligible −2.9% (Figure 2). The values of scavenging activity of compounds **3**,** 5** were lower, and in the case of **3** the decrease in activity was more pronounced. Evidently the activity extent of the compounds tested depends on their molecular structures. The HO-group of 2,6-di-*tert*-butylphenol is the key site in the molecule that is involved in hydrogen transfer to DPPH. However, despite the presence of ferrocene moiety in all the compounds they differ significantly containing linkers of various length and conjugation ability (–CH=N–, **1**; –CH=N–CH_2_–, **2**; –CH=CH–C(O)–, **3**). The decrease of conjugation in their molecules containing either CH_2_ or CO groups in linkers leads to the decrease of metal influence on the stability of radicals formed as it has been observed previously [6]. However, it should be mentioned that the activity of **3 **is much higher than that of **5 **with N atom possessing a lone electron pair in linker that improves the influence of ferrocene moiety. 

To compare the activity of compounds under investigation with that of widely known antioxidant parameter, EC_50_ was determined for the more efficient ferrocene **1** and 2,6-d-*tert*-butyl-4-methylphenol (BHT). EC_50 _values after 30 minutes of experiment at 20°C for **1** and BHT are 34.6 and 105.4 *μ*M, respectively. Therefore, the result obtained shows a more pronounced effect of ferrocenyl derivative of 2,6-di-*tert*-butylphenol. 

In order to study the antioxidant effect of ferrocenes **1**–**6** in biologically significant in vitro test system, we have investigated the compounds influence on Fe^3+^-induced peroxidation of brain homogenate lipids (LP) as a nonenzymatic process by addition of (NH_4_)Fe(SO_4_)_2_. The level of LP was followed by the accumulation of products that reacted with thiobarbituric acid—TBARS. The samples of Wistar strain rats homogenates were divided as following: one control homogenate and samples of homogenate with addition of compounds under investigation. TBARS concentrations were determined in homogenates by measuring the intensity of the solution color at 530 nm using UV-VIS spectroscopy [23]. 

**Figure 3:**
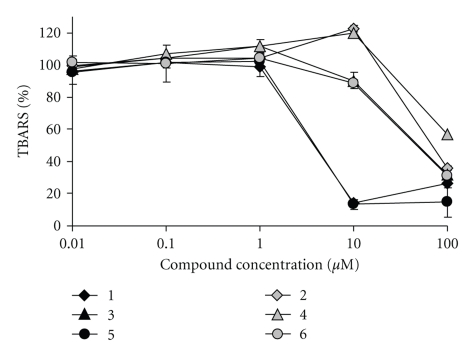
The relative content of TBARS in the lipid peroxidation of rat brain homogenates as nonenzymatic process in the presence of 10 *μ*M **1**–**6** (0.5 mM Fe(NH_4_)(SO_4_)_2_).

The data of antioxidative activity assay of **1**–**6** presenting the pairs of compounds bearing either 3,5-di-*tert*-butyl-4-hydroxyphenyl or phenyl substituents linked to the ferrocene by various spacers are shown in Figure 3. The IC_50_ values are summarized in Table 1. 

The data of antioxidative activity assay of ferrocenes **1**–**6 **indicate the influence of 2,6-di-*tert*-butylphenol group as it was observed in DPPH test. Ferrocene **1** performs an effective inhibitory action in concentrations range at 10–100 *μ*M (Figure 3, curve 1). The decrease in peroxidation level is more that 10%. 

In contrast to DPPH test, the data of this assay reveal the antioxidant activity of all studied compounds. These results allow us to suggest that the ferrocene moiety participates in antioxidative potential of these compounds. However, the compounds **1**,** 3**,** 5** bearing 2,6-di-*tert*-butylphenol are significantly more active than the corresponding phenyl analogues. This effect is most obvious at concentration 10 *μ*M of compounds (Figure 3). Moreover, at this concentration some pro-oxidant effect of compounds **2** and **4** could be observed. This fact might be associated with the influence of iron center in the molecules of ferrocenes that participates in redox processes and therefore promotes the peroxidation. 

The involvement of ferrocene group in the peroxidation process might be associated with the oxidation of Fe^2+^ to Fe^3+^ in the oxidative medium that leads to the formation of ferrocenium cation. As it was proved earlier [24] ferrocenium cations react easily with molecular oxygen and produce reactive peroxy radical cations. On the other hand, in the presence of antioxidant, namely BHT, ferrocenium cation can be stabilized due to the reduction that takes place between the cation and antioxidant. The principal consequence of this electron/proton coupled reaction is the reversibility of ferrocene/ferrocenium redox system. This fact might support the proposition of the intramolecular redox process in ferrocene species containing 2,6-di-*tert*-butylphenol fragment (compounds **1**, **3**, **5**).

To study proapoptotic/antiapoptotic effect of ferrocene **1** with 2,6-d-*tert*-butylphenol group which shows the more promising activity in both tests and to compare it with the effect of its analog **2** bearing phenyl substituent, we have investigated the influence of these compounds on two main characteristics of mitochondria: calcium-induced mitochondrial swelling (SW) that represents the mitochondrial permeability pores opening (which causes cell death), and mitochondrial membrane potential. 

**Figure 4:**
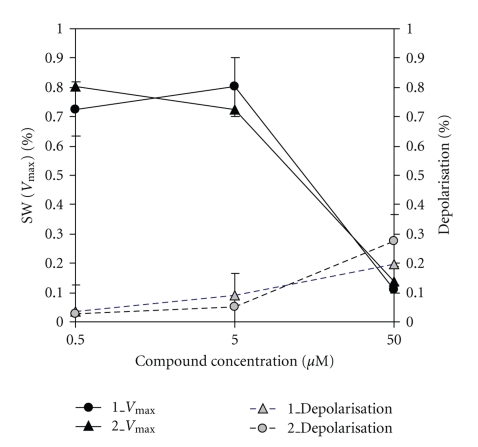
Influence of 0.1 mM ferrocenes **1 **and **2 **on mitochondrial swelling and transmembrane potential (the values were determined and expressed as % of control).

It was shown that at concentration 0.1 mM ferrocenes **1** and **2** slightly depolarize the mitochondria (up to 25%) (Figure 4). On the other hand, these compounds inhibit the calcium-dependent swelling of mitochondria and this effect could not be the consequence of the depolarisation only. In both cases the effects on mitochondrial swelling and mitochondrial membrane potential obtained for ferrocene **1** are less pronounced that for **2**. These data allow us to propose the potential cytoprotective (neuroprotective) effect of compounds studied.

## 4. Conclusion

The antioxidative activity of ferrocenes bearing either 2,6-di-*tert*-butylphenol or phenyl groups, studied using DPPH test, depends strongly upon the presence of phenol group and the conjugation between penoxyl radical formed and ferrocene unit. The compounds **1**,** 3**,** 5** bearing 2,6-di-*tert*-butylphenol are significantly more active than the corresponding phenyl analogues in the in vitro lipid peroxidation in rat brain homogenate. N-(3,5-di-*tert*-butyl-4-hydroxyphenyl)-iminomethylferrocene (**1**) performs a promising behavior as an antioxidant and inhibits the calcium-dependent swelling of mitochondria. The results allow us to propose the potential cytoprotective (neuroprotective) effect of ditopic compounds containing antioxidant 2,6-di-*tert*-butylphenol group and redox active ferrocene fragment.

## Figures and Tables

**Table 1:** The values of IC_50_ in the antioxidative activity assay in rat brain homogenates for compounds **1**–**6**.

Compound	IC_50_ *μ*M (0.5 mM Fe(NH_4_)(SO_4_)_2_)
1	3.7 ± 1.0
2	70.4 ± 11.1
3	47.3 ± 2.4
4	100 ± 15.0
5	3.9 ± 1.8
6	47.8 ± 1.6

## Abbreviations

BHT: butylated hydroxytoluene (2,6-di-*tert*-butyl-4-methylphenol)
DPPH: 1,1-diphenyl-2-picrylhydrazyl (*α*,*α*-diphenyl-*β*-picrylhydrazyl)
HBS: HEPES buffered saline
LP: lipid peroxidation
MPT: mitochondrial permeability transition
RBH: rat brain homogenate
RLM: rat liver mitochondria
TBA: thiobarbituric acid
TBARS: thiobarbituric acid reactive substances.

## References

[1] Migliore L, Coppedè F (2009). Environmental-induced oxidative stress in neurodegenerative disorders and aging. *Mutation Research*.

[2] Kozlowski H, Janicka-Klos A, Brasun J, Gaggelli E, Valensin D, Valensin G (2009). Copper, iron, and zinc ions homeostasis and their role in neurodegenerative disorders (metal uptake, transport, distribution and regulation). *Coordination Chemistry Reviews*.

[3] Crichton RR, Ward RJ (2006). *Metal-Based Neurodegeneration. From Molecular Mechanisms to Therapeutic Strategies*.

[4] Denisov E (1995). *Handbook of Antioxidants*.

[5] Niki E, Yoshida Y, Saito Y, Noguchi N (2005). Lipid peroxidation: mechanisms, inhibition, and biological effects. *Biochemical and Biophysical Research Communications*.

[6] Meleshonkova NN, Shpakovsky DB, Fionov AV, Dolganov AV, Magdesieva TV, Milaeva ER (2007). Synthesis and redox properties of novel ferrocenes with redox active 2,6-di-*tert*-butylphenol fragments: the first example of 2,6-di-*tert*-butylphenoxyl radicals in ferrocene system. *Journal of Organometallic Chemistry*.

[7] Jaouen G (2006). *Bioorganometallics. Biomolecules, Labeling, Medicine*.

[8] van Staveren DR, Metzler-Nolte N (2004). Bioorganometallic chemistry of ferrocene. *Chemical Reviews*.

[9] Stepnicka P (2008). The bioorganometallic chemistry of ferrocene. *Ferrocenes: Ligands, Materials and Biomolecules*.

[10] Hillard E, Vessières A, Thouin L, Jaouen G, Amatore C (2005). Ferrocene-mediated proton-coupled electron transfer in a series of ferrocifen-type breast-cancer drug candidates. *Angewandte Chemie International Edition*.

[11] Vessières A, Top S, Beck W, Hillard E, Jaouen G (2006). Metal complex SERMs (selective oestrogen receptor modulators). The influence of different metal units on breast cancer cell antiproliferative effects. *Dalton Transactions*.

[12] Vessières A, Top S, Pigeon P (2005). Modification of the estrogenic properties of diphenols by the incorporation of ferrocene. Generation of antiproliferative effects in vitro. *Journal of Medicinal Chemistry*.

[13] Qiu X, Zhao H, Lan M (2009). Novel ferrocenyl nitroxides: synthesis, structures, electrochemistry and antioxidative activity. *Journal of Organometallic Chemistry*.

[14] Mugesh G, Panda A, Singh HB, Punekar NS, Butcher RJ (1998). Diferrocenyl diselenides: excellent thiol peroxidase-like antioxidants. *Chemical Communications*.

[15] Milaeva ER, Gerasimova OA, Jingwei Z (2008). Synthesis and antioxidative activity of metalloporphyrins bearing 2,6-di-*tert*-butylphenol pendants. *Journal of Inorganic Biochemistry*.

[16] Tyurin VYu, Gluchova AP, Meleshonkova NN, Milaeva ER Electrochemical method of antioxidative activity assay based on DPPH test.

[17] Brand-Williams W, Cuvelier ME, Berset C (1995). Use of a free radical method to evaluate antioxidant activity. *Food Science and Technology*.

[18] Molyneux P (2004). The use of the stable free radical diphenylpicrylhydrazyl (DPPH) for estimating antioxidant activity. *Songklanakarin Journal of Science and Technology*.

[19] Serkov IV, Shevtsova EF, Dubova LG (2007). Interaction of docosahexaenoic acid derivatives with mitochondria. *Doklady Biological Sciences*.

[20] Gornall AG, Bardawill CJ, David MM (1949). Determination of serum proteins by means of the biuret reaction. *Journal of Biological Chemistry*.

[21] Callaway JK, Beart PM, Jarrott B (1998). A reliable procedure for comparison of antioxidants in rat brain homogenates. *Journal of Pharmacological and Toxicological Methods*.

[22] Foti MC, Daquino C, Mackie ID, DiLabio GA, Ingold KU (2008). Reaction of phenols with the 2,2-diphenyl-1-picrylhydrazyl radical. Kinetics and DFT calculations applied to determine ArO-H bond dissociation enthalpies and reaction mechanism. *Journal of Organic Chemistry*.

[23] Paquot C, Hantfenne A (1987). *Standard Methods for the Analysis of Oils, Fats and Derivatives*.

[24] Hurvois JP, Moinet C (2005). Reactivity of ferrocenium cations with molecular oxygen in polar organic solvents: decomposition, redox reactions and stabilization. *Journal of Organometallic Chemistry*.

